# Two-Step Synthesis of CuS/C@PANI Nanocomposite as Advanced Electrode Materials for Supercapacitor Applications

**DOI:** 10.3390/nano10061034

**Published:** 2020-05-28

**Authors:** Qing Liu, Shihang Zhang, Yan Xu

**Affiliations:** 1 Department of Electric Engineering, North China Electric Power University, Baoding 071003, China; 51351103@ncepu.edu.cn (Q.L.); xuyanhd@ncepu.edu.cn (Y.X.); 2 State Key Laboratory of New Energy and Electric Power Systems, North China Electric Power University, Baoding 071003, China

**Keywords:** CuS/C-120@PANI, nanocomposites, supercapacitor, electrode material

## Abstract

In this study, the dense cloud-like structured CuS nanoparticles were successfully prepared using a simple two-step hydrothermal method. The experimental temperature was the most important factor that affected the microstructure and surface functions of CuS/C. Therefore, the CuS/C electrodes were synthesized at different temperatures (80 °C, 120 °C, and 160 °C). Subsequently, their crystallographic phase and morphologies as well as the structure of the as-prepared electrodes were analyzed in detail. The electrode prepared at 120 °C (CuS/C-120) was determined to have a perfect microstructure, high specific capacitance, and good rate performance. To further improve the electrochemical performance of this electrode, it was combined with polyaniline (PANI) to obtain a CuS/C-120@PANI electrode via the cyclic voltammetric electrodeposition method. The CuS/C-120@PANI electrode exhibits a specific capacitance of 425.53 Fg^−1^ at a current density of 1 Ag^−1^ and a good cycling stability of 89.86% after 3000 cycles. The perfect architecture of CuS/C-120@PANI maximizes the synergistic effect between its different components and provides abundant electrochemically reactive sites, promoting the diffusion and transfer of electrolyte ions during the electrochemical reaction processes. Detailed analysis shows that the CuS/C-120@PANI electrode has great potential for use in high-performance energy storage devices.

## 1. Introduction

Owing to the heavy consumption of fossil fuels and the increasing environmental pollution, renewable energy power generation technologies, such as wind, solar, and tidal energy have rapidly developed. Further, new energy conversion and storage devices are urgently required. Supercapacitors (SCs) exhibit considerable potential for use in electrical equipment and other applications because they can quickly store energy and effectively provide electrical energy [1,2,3]. When compared with batteries, SCs have the advantages of a large energy density, long cycle life, and fast charge–discharge rates [4,5]. According to the charge storage mechanism, the energy storage of SCs can be divided into two types based on the ion adsorption at the electrode–electrolyte interface or the reversible redox reaction on the electrode surface. Carbon materials are generally used as electrode materials in double-layer capacitors owing to their good cycle stability. However, they have a low energy density [6]. Transition metal oxides and conductive polymers are used in pseudocapacitors owing to their high specific capacitances [7,8]. SCs experience a decrease in cycle life owing to the occurrence of the Faraday redox reaction [9]. Therefore, the development of new high-quality and environment-friendly electrode materials for SCs is the key to optimize their excellent performance [10].

Recently, electrode materials, such as cobalt sulfide [11] and molybdenum sulfide [12], have been widely used in SCs owing to the unique physical and chemical properties exhibited by transition metal sulfides and oxides in the field of energy storage. CuS is an important semiconductor material that is easy to synthesize. Further, it can be prepared from extensively used abundant raw materials, making it an ideal electrode material. Li et al. synthesized a streamlined CuS nanoarray with respect to graphene nanosheets [13]. Liang et al. prepared CuS via a facile hydrolysis method with a graphene multilayered two-dimensional (2D) structure [14]. Huang et al. synthesized a CuS/multi-walled carbon nanotube (MWCNT) composite electrode material with a hierarchical structure via a step hydrothermal method [15]. However, it has a relatively low conductivity when compared with carbon materials. Therefore, it is highly desirable to study the CuS composites with an electronically conductive substance.

Based on the previous research results, CuS/C electrodes were synthesized in this study via a two-step hydrothermal method to improve its conductivity. The metal–organic framework (MOF) materials exhibit the advantages of good thermal stability, large specific surface area, and adjustable pore size [16,17,18]. MOFs have unique structures and contain suitable channels that allow the passage of electrolytes. Further, a conductive material that can transport charges can be added into the framework, making MOFs suitable templates to prepare CuS/C electrode materials. Therefore, an MOF template can be used to effectively prepare CuS/C electrode materials. The performance of any SC electrode material is dependent on many parameters, including the experimental temperature, reaction time, and actual duty cycle [19,20]. Thus, the electrochemical performance is affected by the synthesis and properties of the nanostructured electrode material. Hence, this study focuses on the experimental temperature. Furthermore, the conductivity of electrode materials can be improved by conducting polymers. Polyaniline (PANI) is a promising conducting polymer in the energy storage field because of its abundance, high specific capacitance, and high electron conductivity (30–200 S cm^−1^) compared with those of other polymers [21,22]. Further, PANI can achieve oxidation–reduction reactions in various states using electrochemical methods. PANI exhibits good dispersibility and structural integrity when compared to polyacetylene; therefore, it is considered to be a highly promising material for energy–storage applications [23]. Recently, the synthesis and supercapacitor properties of PANI composites have been studied. Zhu et al. [24] constructed a hierarchical ZnO@MOF@PANI on carbon cloth for supercapacitor electrodes. The specific capacitance of ZnO@MOF@PANI is as high as 340.7 Fg^−1^ at a current density of 1 Ag^−1^. Jafari et al. [25] synthesized MOF-199 and the composite HKUST-1/PANI. The specific capacitance of HP can approach to be 270 Fg^−1^ at 1 Ag^−1^. Wang et al. had reported the PANI-ZIF-67-CC electrode with a high specific capacitance of 2146 mF/cm^2^ at 10 mVs^−1^ [26]. The composites containing several kinds of materials can show the advantages of different electrode materials. Furthermore, the positive synergy of each electrode material can improve the electrochemical performance. Based on the above discussion, the highly conductive CuS/C@PANI nanocomposite electrode material is expected to be an excellent electrode in energy storage devices. To the best of our knowledge, the composite materials comprising PANI and copper sulfide for supercapacitors have been rarely reported.

In this study, CuS/C electrodes were synthesized at different temperatures (80 °C, 120 °C, and 160 °C), and their morphologies were compared; furthermore, their electrochemical performances were investigated in a 3 M KCl neutral electrolyte. A CuS/C-120@PANI nanocomposite that exhibits a high specific capacitance and the best cycling stability was subsequently prepared at a curing temperature of 120 °C. When compared with other studies, the CuS/C-120@PANI synthesized in this study exhibited two advantages. First, it has a unified and highly ordered special framework owing to the use of a Cu–MOF as the precursor. Second, it exhibits good cycling stability and high rate capability owing to the addition of the conductive polymer PANI.

## 2. Materials and Methods

### 2.1. Materials

All the chemicals were used as received. Copper nitrate trihydrate (Cu(NO_3_)_2_·3H_2_O, 99.99%) was supplied by Tianjin Tianli Chemical Co., Ltd. (Tianjin, China), 1,3,5-benzenetricarboxylic acid (H_3_BTC, 95%), polyvinyl pyrrolidone (PVP, 99%), thioacetamide (CH_3_CSNH_2_, 99%), aniline (PANI, 99.5%), potassium chloride (KCl, 99.5%), and 1-methyl-2-pyrrolidinone (NMP, 99%) were acquired from Aladdin, and solvents (N, N-dimethylformamide and ethanol with 99.98% and 99% of purity, respectively) were obtained from Aladdin Company (Shanghai, China). All the chemicals were of the analytical-reagent grade and used as received without any further purification. The solutions were prepared using ultrapure water (18.2 MΩ·cm) throughout the experiments. Hydrophobic carbon cloth (CC, 1 cm × 2 cm, WOS1002) was provided by CeTech Co. Ltd., Taiwan, China.

### 2.2. Synthesis of the CuS/C-80/120/160 Materials

To prepare the CuS/C-based electrode materials used in this study, the first step was to prepare a Cu–MOF precursor [27]. Cu(NO_3_)_2_·3H_2_O (0.7248 g) and H_3_BTC (0.4242 g) were dissolved in 12 mL of 9:3 (*v*/*v*) dimethylformamide:ethanol (DMF:EtOH) under ultrasonication and magnetic stirring for 40 min, respectively. Then, the BTC solution was added dropwise to the Cu^2+^ solution under rapid stirring. Once the two solutions were mixed, 0.1 g of polyvinyl pyrrolidone (PVP) was added dropwise to the solution as a surface stabilizer. Subsequently, the reaction mixture was sealed in a 100 mL Teflon-lined stainless steel autoclave and heated in a water bath at 140 °C for 24 h. After the completion of the reaction, the sample was allowed to naturally cool to room temperature and subsequently washed thrice using EtOH. Finally, the resulting solid powder was annealed under argon atmosphere at 350 °C for 12 h to obtain a black powder.

The next stage was to prepare the CuS/C electrode materials. Three sets of the synthesized Cu–MOF (0.0800 g) and thioacetamide (0.1800 g) of equal mass were weighed and dissolved in 20 mL of ethylene glycol. The particles were subsequently dispersed using an ultrasonic oscillator to obtain homogeneous aqueous solutions. Furthermore, the two solutions were mixed together and evenly stirred, resulting in the immediate appearance of a bluish-green precipitate. After the mixtures were stirred for 30 min, they were transferred to Teflon-lined stainless steel autoclaves and placed in three identical vacuum drying ovens set to a temperature gradient to heat for 12 h. The experimental temperatures were 80 °C, 120 °C, and 160 °C. After the vulcanization reaction, the samples were allowed to cool to room temperature, and they were subsequently washed thrice using EtOH. Finally, the resulting black products indicated the formation of CuS/C. The samples were labeled as CuS/C-80, CuS/C-120, and CuS/C-160 based on the vulcanization temperature considered in each case. During the experiments, all the parameters, except the experimental temperature, were maintained unchanged. Therefore, the experimental temperature can be considered for comparison purposes.

### 2.3. Fabrication of the CuS/C-120@PANI Electrode Electrodes

**Figure 1:**
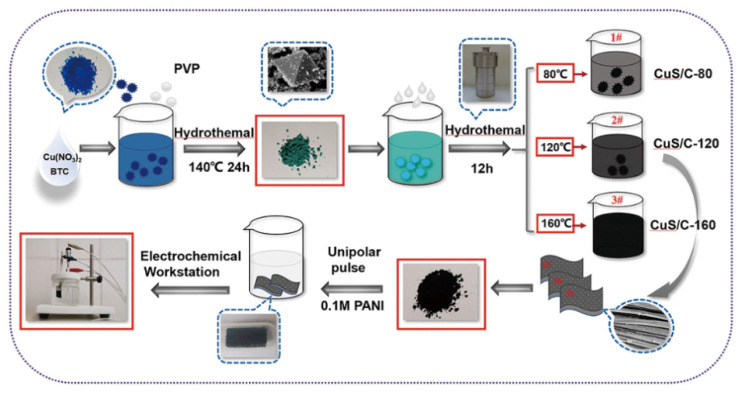
Schematic illustration of the fabrication of CuS/C-120@PANI nanocomposite.

PANI was deposited on the newly prepared CuS/C-120 electrode in the freshly prepared 3 M KCL solution containing 0.1 M PANI via a cyclic voltammetric electrodeposition [28], which was performed in a three-electrode system from −0.3 to 0.6 V at 10 mV^−1^ for 20 cycles. Here, the Ag/AgCl and Pt foil were applied as the reference electrode and counter electrode. When the deposition was completed, the electrode was gently rinsed using distilled water and dried prior to use. The specific experimental process is presented in Figure 1.

### 2.4. Morphological and Structural Characterization

The crystal structure of the samples was identified through X-ray diffraction (XRD) measurements recorded on an X-ray diffractometer (Bruker D8, Rigaku, Tokyo, Japan, Cu Kαradiation, λ = 0.15406 nm). The microstructure of the samples was observed with FESEM (HITACHI, S4800, Tokyo, Japan) and TEM (JEOL, 2100F, Tokyo, Japan). Fourier transform infrared spectroscopy spectra were conducted by an FTIR spectrometer (Thermo Nicolet 5700 system, Hitachi, Berlin, Germany). XPS (Thermo ESCALAB 250Xi, Shanghai, China) was performed to identify surface com-position of ternary composite inside an ultrahigh vacuum system with an excited MtgKα source (1486.6 eV). Raman spectrum was obtained using a 514 nm excitation laser with a WITec Raman instrument (Renishaw PLC, London, United Kingdom). In addition, the specific surface area of as-fabricated samples was evaluated by utilizing Brunauer-Emmett-Teller analysis (TriStar 3020, Micromeritics, New York, NY, USA).

### 2.5. Electrochemical Analysis

The working electrodes were prepared by mixing 80% active materials, 15% polyvinylidene fluoride, and 5% acetylene black. Then, NMP was added dropwise and stirred to prepare slurry. The resulting slurry was coated onto the CC, which was followed by drying at 60 °C for 12 h in a vacuum oven.

The electrochemical performance of all electrodes was performed with the electrochemical workstation (CHI 660E, Chenhua, China) in a typical three-electrode system with 3 M KCL aqueous solution as the electrolyte. Pt, Ag/AgCl, and CuS/C electrodes were operated as the counter, reference, and working electrodes, respectively. All the electrochemical test including cyclic voltammetry (CV) and galvanostatic charge–discharge (GCD) were carried out from −0.3 to 0.6 V. The electrochemical impedance spectroscopy (EIS) test was observed within a frequency range from 10^−2^ to 10^5^ Hz with an amplitude of 5 mV. The cycling activity was evaluated by a continuous cyclic voltammetry process at a scan rate 10 mV^−1^ for over 3000 cycles. The specific capacitance was calculated from the CV cures and GCD curves according to the following equation [29]:

(1) $$C=\frac{\int I\cdot dV}{2v\cdot m\cdot ∆V}$$

(2) $$C=\frac{I\cdot ∆t}{m\cdot ∆V}$$

where *I* (A) is the charge-discharge current, *m* (g) is the weight of active materials on electrode, *t* (s) is the discharge time, *V* (V) is the potential window, and *v* (mV^−1^) is the scan rate.

## 3. Results Discussion

### 3.1. Morphological and Structural Characterization

**Figure 2:**
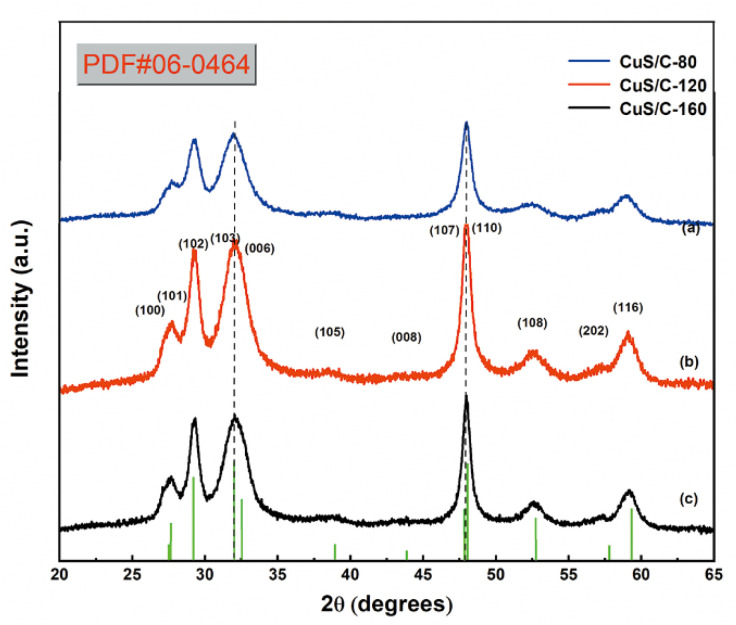
Powder X-ray diffraction (PXRD) patterns of (**a**) CuS/C-80, (**b**) CuS/C-120, and (**c**) CuS/C-160 nanoparticles.

The structures of CuS/C-80, CuS/C-120, and CuS/C-160 were investigated based on the powder X-ray diffraction (PXRD) measurements. The PXRD patterns of the CuS/C samples at different experimental temperatures are presented in Figure 2, where the diffraction peaks of the three patterns at 2θ = 27.223°, 27.738°, 29.323°, 31.914°, 38.915°, 48.016°, 52.777°, and 59.428° can be assigned to the (100), (101), (102), (103), (105), (110), (108), and (116) planes of CuS (PDF NO.06-0464), respectively. No diffraction peaks could be observed from other substances, indicating that the synthesized CuS samples do not contain impurities. When compared with CuS/C-80 and CuS/C-160, the PXRD pattern of CuS/C-120 shows the sharpest peaks, implying that carbon in the composites obtained at 120 °C had a higher degree of crystallization. Additionally, the CuS crystallite size were calculated via a Scherrer equation [30]:

(3) $$D=\frac{K\lambda }{\beta cos\theta }$$

where “*K*” is the shape factor having constant value 0.9, “*λ*” is X-rays wavelength (1.541874 Å), “*β*” is the full width at half maximum (FWHM) of the high-intensity peak and *θ* is the Bragg’s angle of the radians. The average crystallite size of the CuS were 8.1 nm, 9.7 nm, and 8.4 nm for CuS/C-80, CuS/C-120, and CuS/C-160, respectively. Therefore, CuS/C-120 has the largest crystallite size and the highest degree of crystallization, as shown in Table 1. CuS/C exhibits strong diffraction peaks at low angles, indicating the presence of abundant of pores in the sample. Therefore, the crystallization of the composites changes upon an increase in the hydrothermal temperature, which may affect their electrochemical performance.

**Figure 3:**
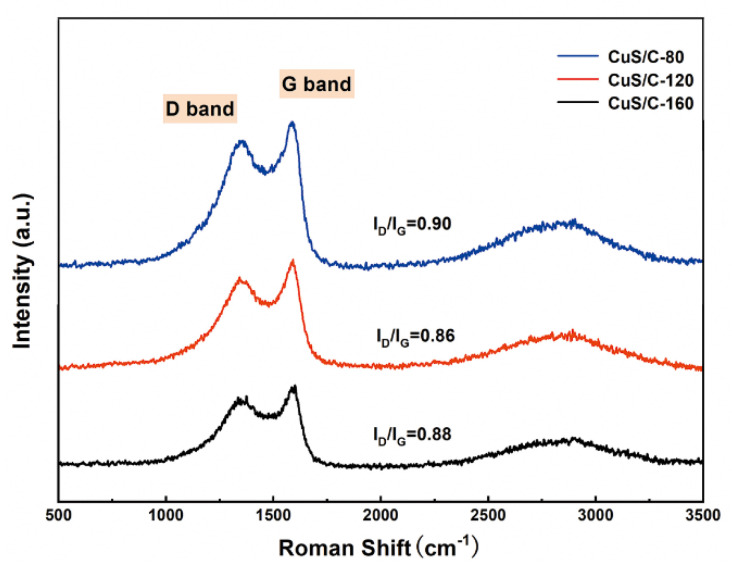
Raman spectra of CuS/C-80, CuS/C-120, and CuS/C-160 nanoparticles.

The graphitized structures of the CuS/C samples at different temperatures were further analyzed using Raman spectroscopy, the results of which are shown in Figure 3. In the Raman spectra, two intense peaks can be observed at 1342.5, 1585 cm^−1^ and 1585 cm^−1^, which can be assigned as the D and G bands, respectively. The presence of the D band peak can be attributed to the disordered structure of the prepared carbon electrode material, whereas the G band peak represents the vibration of the sp^2^ hybridized carbon atoms. The I_D_ to I_G_ ratio indicates the degree of graphitization in the structure. The measured values of I_D_/I_G_ were 0.90, 0.86, and 0.88 for CuS/C-80, CuS/C-120, and CuS/C-160, respectively. The smaller the ratio, the higher will be the degree of graphitization and the more disordered will be the structure. The I_D_/I_G_ of CuS/C-120 is lower compared to CuS/C-80 and CuS/C-160, expressing that the graphitic crystalline structure of CuS/C-120 is superior to that of CuS/C-80 and CuS/C-160. In another word, it indicates that the CuS/C-80 and CuS/C-160 possess higher disordered graphitic structure than CuS/C-120. At the same time, the increase in the intensity of the D-band of CuS/C-80 and CuS/C-160 revealed the presence of additional crystal defects and structural disorderness. However, the CuS/C-120 exhibits the lower I_D_/I_G_ specific value than that of CuS/C-80 and CuS/C-160, which is due to the reduction of disordered graphitic structure of the surface when the temperature is 120 °C. This indicates that the CuS/C-120 structure is more conducive for the transportation of internal electrons, increasing the conductivity of the material. As the experimental temperature increases, the degree of graphitization initially increases and subsequently decreases, indicating that the graphite crystal structure of CuS/C-120 is the best among the three samples.

**Figure 4:**
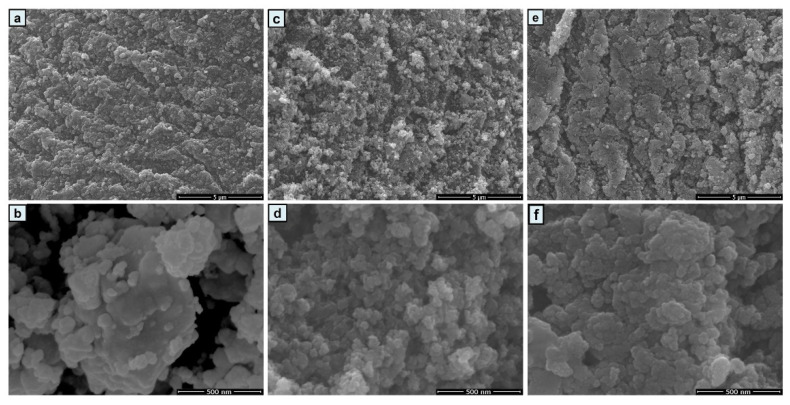
Low and high magnification SEM images of (**a**,**b**) CuS/C-80, (**c**,**d**) CuS/C-120, and (**e**,**f**) CuS/C-160.

The morphologies and microstructures of the CuS/C-80, CuS/C-120, and CuS/C-160 samples were characterized using scanning electron microscopy (SEM, Figure 4a–f). When the experimental temperature reaches 80 °C, the granulation in the sample is manifest. When the temperature increases from 120 to 160 °C, the nanoparticles are connected into a block. Further, when the temperature is 120 °C, they collide with other nanoparticles to form a dense cloud-like nanostructure. Compared with CuS/C-160 and CuS/C-80, CuS/C-120 exhibits a more regular flocculent network structure, with almost no agglomeration and a smoother surface. The homogeneous nanoparticles in the sample contain gaps between them. These gaps are beneficial for the diffusion and transfer of the electrolyte ions, which enhances the conductivity of the samples, endowing it with the best electrochemical properties among the samples. Therefore, temperature is the key factor that affects the structure of the materials.

**Figure 5:**
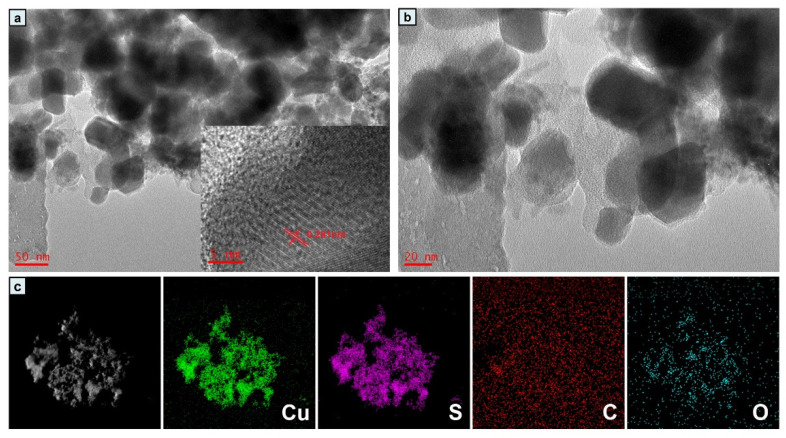
(**a**,**b**) TEM images of CuS-120 nanoparticles; (**c**) EDX mapping of Cu, S, C, O for CuS-120.

The microstructure of the CuS/C-120 composite was examined using TEM. It can be seen from the images in Figure 5a,b that the CuS/C-120 nanometer-sized dense spatial cloud structure is clearly hierarchical. Further, the CuS/C nanoparticles in the sample comprise elemental Cu, S, and C, and C is embedded in the center of CuS/C. Polyhedral crystals of approximately 60 nm in size can be observed, and the CuS/C nanoparticles are interconnected. The high-resolution transmission electron microscope image in Figure 5a shows lattice fringes with a distance of 0.281 nm, which could be attributed to the (103) crystal plane. To confirm the coexistence of each element, the energy dispersive X-ray spectrometer coupled with the microscope was used to perform elemental mapping analysis, the results of which are shown in Figure 5c. SEM and TEM analysis showed that the internal structure of CuS/C is regulated by the experimental temperature. This nanostructure can be used to achieve convenient electron transport, which is helpful with respect to the electrochemical performances.

**Figure 6:**
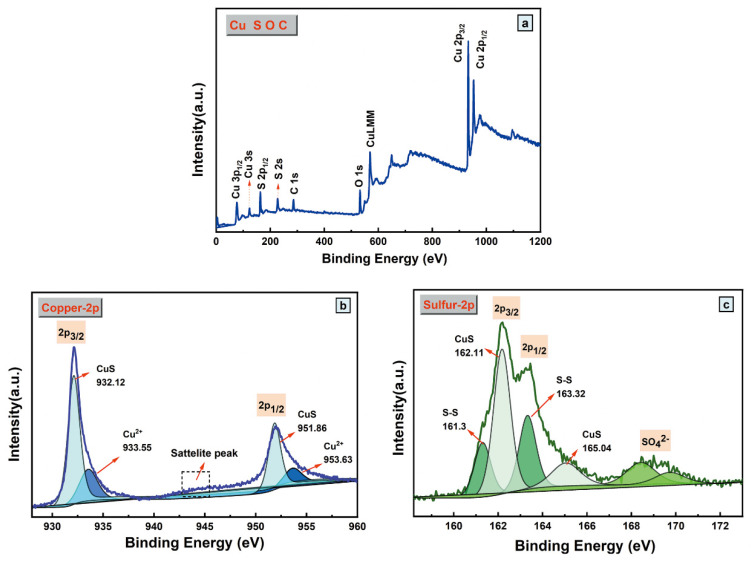
(**a**) Full XPS patterns of CuS/C-120; XPS patterns of (**b**) Cu 2p and (**c**) S 2p.

X-ray photoelectron spectroscopy (XPS) measurements were conducted to determine the elemental composition of CuS/C-120, and their results are presented in Figure 6. The spectrum exhibits signals containing Cu, S, C, and O elements, and there is no other impurity element, as presented in Figure 6a. The Cu 2p spectrum is shown in Figure 6b. The main peaks located at 932.12 eV and 951.86 eV in the spectrum correspond to Cu 2p_3/2_ and Cu 2p_1/2_, respectively [31]. The peaks at 932.12 eV and 951.68 eV could be attributed to Cu^2+^ in CuS [32], whereas those at 933.55 eV and 953.63 eV can be fitted to Cu^2+^ in CuSO_4_^2−^ [33]. Figure 6c shows S 2p, where there are four main peaks at 162.17 eV and 163.71 eV, which can be attributed to S 2p_3/2_ and S 2p_1/2_, respectively, and 162.11 eV and 165.04 eV, which can be assigned to the elemental S in CuS [34]. In the same spectrum, the peaks at 161.3 eV and 163.32 eV can be assigned to the S–S dimers [35], and the peak at 168.65 eV is characteristic of SO_4_^2−^ [36], which is present on the surface of CuS/C-120 because of the partial air oxidation of the sample surface. The XPS results denote that CuS/C-120 mainly comprises Cu^2+^ and S^2−^.

**Figure 7:**
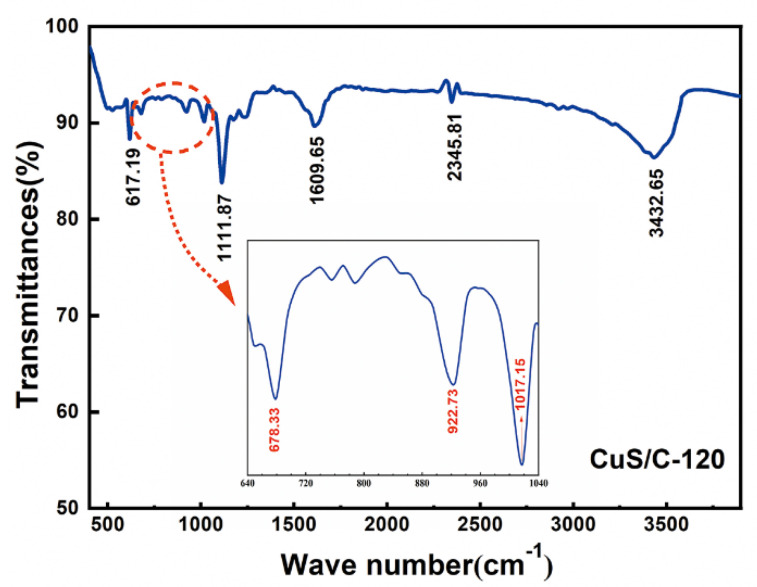
Fourier transform infrared spectroscopy (FTIR) spectra of the CuS/C-120 nanoparticles.

Fourier transform infrared (FTIR) spectroscopy is performed to investigate the compositions of the functional groups on the surface of the CuS/C-120 electrode material, and the results are presented in Figure 7. Five main absorption peaks can be observed in the spectra at 617.19, 1111.87, 1609.65, 2345.81, and 3432.65 cm^−1^. The absorption peaks between 617.19 and 1111.87 cm^−1^ are shown in more detail in the inset in Figure 7. The absorption peaks at 1111.87, 1609.65, and 2345.81 cm^−1^ can be attributed to the C–OH, C=O, and C=C stretching vibrations, respectively. The absorption peak at 3432.65 cm^−1^ can be attributed to the stretching of the -OH in the carboxyl group, providing the presence of bound H_2_O or ethanol molecules after the two-step hydrothermal reaction [37,38].

**Figure 8:**
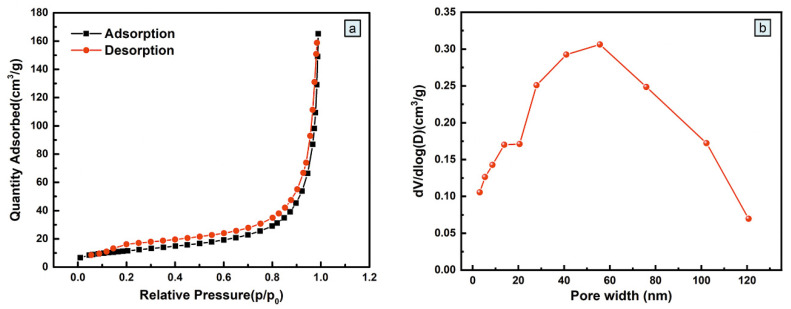
(**a**) N_2_ adsorption/desorption isotherms for the CuS/C-120 electrode material; (**b**) Pore size distribution of the CuS/C-120 electrode material.

The specific surface area and pore size of a material are closely related to its electrochemical performance. Therefore, the specific surface area and pore size distribution of CuS/C-120 were measured and analyzed using the N_2_ adsorption–desorption isotherms at 77.350 K, as shown in Figure 8a, where the isotherms are type-IV with a H_3_ hysteresis loop. The hysteresis loop is obvious when the relative pressure is p/p_0_ > 0.3. When p/p_0_ > 0.9, the upward shift in N_2_ isotherm could be attributed to the existence of perforations in the sample. When p/p_0_ < 0.1, there is no obvious upward shift in the isotherms, indicating that there are very few micropores in the sample. These results indicate that CuS/C-120 mostly exists as mesopores and macropores, forming a layered porous structure. In the initial stage of CuS formation, with the progress of the sulfidation reaction, the amount of Cu^2+^ required for CuS formation increased, and the volume of CuS nanoparticles gradually increased with the progress of sulfidation reaction. Pores were formed when ions passed through the CuS particles from the gap under the electric field force [39]. As the reaction continued, the pores gradually became larger and transformed into mesopores and macropores. Therefore, micropores, mesopores, and macropores were formed because of the different reaction times and temperatures. Besides, several mesoporous were formed during the carbonization [40]. Micropores can contribute to charge storage, macropores can serve as the “ion buffering reservoirs”, and mesopores can facilitate to penetrate the electrolyte ions into the whole materials [41]. Figure 8b shows the pore size distribution of CuS/C-120, which supports the existence of mesopores (50 nm) in its structure. A porous structure not only allows the rapid penetration of electrolytic ions into the electrode material but also provides several reactive sites for ion diffusion, reducing the ion diffusion distance. The average specific surface area of the prepared CuS/C-120 powder is 41.3709 m^2^/g. The contact area between the electrode material and the electrolyte increases because of a high specific surface area, providing a good environment for the transport of ions and electrons, which is beneficial for redox reactions. Furthermore, a high specific surface area is one of the most important reasons for the excellent electrochemical performance of CuS/C-120. The N_2_ adsorption–desorption isotherm results were consistent with the previous PXRD, SEM/TEM, XPS, FTIR, and Raman spectroscopic analysis results.

### 3.2. Electrochemical Performance

**Figure 9:**
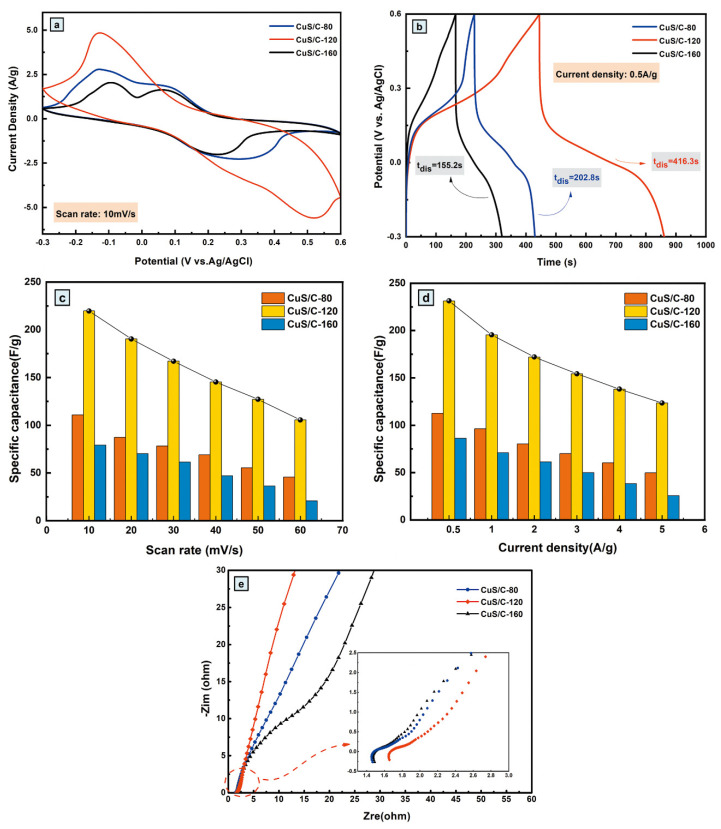
(**a**) Comparison cyclic voltammetry (CV) curves for CuS/C-80, CuS/C-120 and CuS/C-160 electrodes at 10 mV^−1^; (**b**) Comparison charge-discharge curves for CuS/C-80, CuS/C-120, and CuS/C-160 electrodes at 0.5 Ag^−1^; (**c**) The specific capacitance at different scan rates of CuS/C-80, CuS/C-120, and CuS/C-160 electrodes; (**d**) The specific capacitance at different current densities of CuS/C-80, CuS/C-120, and CuS/C-160 electrodes; (**e**) Nyquist electrochemical impedance spectra of CuS/C-80, CuS/C-120, and CuS/C-160 electrodes (inset: the enlarged high frequency part).

Electrochemical test analysis was performed to further explore the effect of temperature on the electrochemical performance of the CuS/C electrode materials and improve the conductivity of the electrode materials at optimal temperatures. The CV curves of CuS/C were recorded in the voltage window of −0.3–0.6 V at a scan rate of 10 mV^−1^ and different hydrothermal temperatures, as shown in Figure 9a. Among the CV curves of the three samples, that of CuS/C-80 exhibits a rectangular shape. The CV curves of CuS/C-120 and CuS/C-160 exhibit obvious redox peaks, indicating the occurrence of the redox reactions. Moreover, the loop area of CuS/C-120 is considerably larger than those of CuS/C-80 and CuS/C-160. Therefore, it exhibits the highest specific capacitance. The specific capacitances of CuS/C-80, CuS/C-120, and CuS/C-160 are 110.76, 219.65, and 79.16 Fg^−1^ at a scan rate of 10 mV^−1^. Figure 9b shows the charge-discharge curves (GCD) of the three samples at a current density of 0.5 Ag^−1^. All three curves are approximately triangular in shape and are stable. Notably, CuS/C-120 has the longest discharge time (416.3 s), which is longer than those of CuS/C-80 (202.8 s) and CuS/C-160 (155.2 s). In addition, the calculated specific capacitances of CuS/C electrodes based on the discharge curves are 112.67, 231.32, and 86.22 Fg^−1^ at temperatures of 80 °C, 120 °C, and 160 °C, respectively. These results are consistent with the CV curves. Figure 9c,d show the histograms of the specific capacitances of the three samples at different current densities and scan rates. With an increase in the current density (scan rate), the specific capacitances of all the three samples decreased, because a high current density limits the rate of ion migration to the interior of the active material. Moreover, CuS/C-120 has the largest specific capacitance at different current densities owing to its internal structure, electrical conductivity, and large specific surface area. Figure 9e shows the alternating current (AC) impedance spectra of the three samples, which is an enlarged view of the high-frequency part of the spectrum. The Nyquist plots of the samples include semicircular and linear regions. Compared with CuS/C-80 and CuS/C-160, CuS/C-120 has the steepest Nyquist plot slope, exhibiting high specific capacitance. Thus, it exhibits superior electrochemical properties owing to its good rate performance, large pore volume, and high specific surface area. The results demonstrate that the capacitance of the composites is considerably dependent on the experimental temperature.

**Figure 10:**
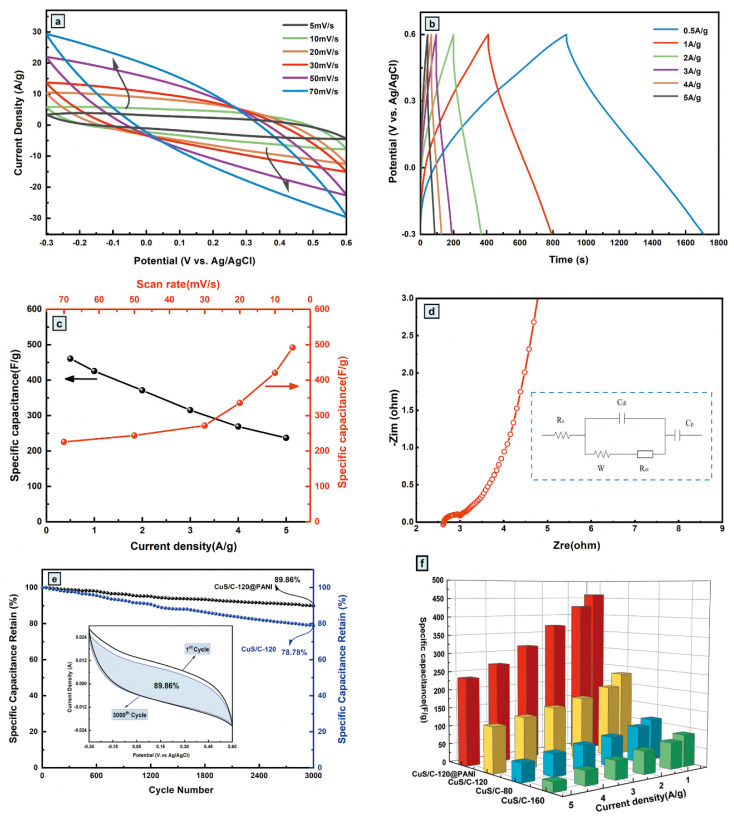
(**a**) CV curves at various scan rates for CuS/C-120@PANI electrode; (**b**) GCD curves at various current densities for CuS/C-120@PANI electrode; (**c**) Specific capacitance values of different various scan rates and current densities; (**d**) EIS curve of CuS/C-120@PANI with equivalent circuit inset; (**e**) Cycling performance of the CuS/C-120@PANI electrode at 10 mV^−1^ for 3000 cycles; (**f**) Specific capacitance of CuS/C-80, CuS/C-120, CuS/C-160 and CuS/C-120@PANI at different current densities.

To improve its electrochemical performance, the conductive polymer polyaniline (PANI) was deposited on CuS/C-120 to obtain a highly conductive CuS/C-120@PANI nanocomposite electrode material. The CV curves of CuS/C-120@PANI at a scan rate of 5–70 mV^−1^ are shown in Figure 10a. It can be observed from the CV scans that the current peak gradually increases with an increasing scan rate, maintaining a similar rectangular shape to that of the CV curves of CuS/C-120, without obvious redox peaks, which indicates that CuS/C-120@PANI exhibited better reversibility when compared with that exhibited by CuS/C-120. The specific capacitance is proportional to the CV integral area; therefore, the CuS/C-120@PANI nanocomposite shows significantly enhanced capacitance, reflecting high charge storage efficiency because of the contribution of polyaniline. To evaluate the CuS/C-120@PANI nanocomposite, the rate performance was also analyzed by using the GCD curves obtained at various current densities. The GCD curves at various current densities are shown in Figure 10b, where the discharge time of the composite electrode gradually decreases from 0.5 to 5 Ag^−1^. Based on Formula (2), the calculated specific capacitances of CuS/C-120@PANI are 460.66, 425.53, 371.16, 315.37, 268.99, and 237.00 Fg^−1^ at discharge current densities of 0.5, 1.0, 2.0, 3.0, 4.0, and 5.0 Ag^−1^, respectively. However, the GCD curves are not deformed, and they remain approximately triangular, implying that the GCD curves are in good agreement with the CV results. The IR drop values obtained under different current densities are presented in Table 2.

The voltage loss is minimum when the current density is the lowest. The specific capacitance of CuS/C-120@PANI obtained under different current densities and scan rates are presented in Figure 10c, exhibiting a good rate capability. The AC impedance spectrum of the composite electrode is presented in Figure 10d. The inset shows the fitting circuit R (C (WR)) C, where R_s_, R_ct_, C_dl_, W, and C_p_ represent the solution impedance, charge transfer impedance, electric double-layer capacitance, Warburg impedance, and faradaic pseudo capacitance, respectively. In the high-frequency region, the R_ct_ value is large, indicating that the porous structure of the composite electrode is conducive to charge transfer. Furthermore, in the low-frequency region, the slope is approximately vertical, indicating that the electrode exhibits an electrochemical behavior approximately similar to pure capacitance. The EIS results confirm the excellent supercapacitive behavior of CuS/C-120@PANI electrode. Figure 10e shows the cyclic stability of the CuS/C-120@PANI after 3000 cycles at a scan rate of 10 mV^−1^. The capacitance retention rate of the electrode became 89.86%, implying good cyclic stability. Figure 10f shows a three-dimensional comparison of the capacitance retention of CuS/C-80, CuS/C-120, CuS/C-160, and CuS/C-120@PANI under different current densities. The CuS/C-120@PANI composite possesses the largest capacitance among them all. The capacitance characteristics are optimal when the hydrothermal temperature is 120 °C at different experimental temperatures. Therefore, the experimental temperature was considered to be the key factor that affects the performance of the electrode material. Further, the combination of PANI with CuS/C-120 enhances its performance. The positive synergistic effects between PANI and CuS/C-120 can enhance the corresponding electrochemical characteristics. CuS/C-120@PANI was the most suitable electrode material for use in SCs and could play an important role in the field of materials.

## 4. Conclusions

In summary, CuS/C electrode materials with an excellent structure were successfully prepared using a two-step hydrothermal method. The curing temperature exhibited an important effect on the structural properties and electrochemical performance of the materials. In a three-electrode test system, the CuS/C-120 electrode exhibited a specific capacitance of 231.32 Fg^−1^ at 0.5 Ag^−1^, which is considerably higher than the values observed for CuS/C-80 and CuS/C-160. To further improve the capacitance performance of CuS/C-120 in terms of conductivity, a CuS/C-120@PANI electrode nanocomposite was prepared by depositing the conductive polymer PANI on the CuS/C-120 electrode, resulting in a specific capacitance of 425.53 Fg^−1^ at 1 Ag^−1^ in 3 M KCL. In addition, the composite electrode retained 89.86% of its capacitance after 3000 cycles. CuS/C-120 exhibited excellent electrochemical performance, which could be attributed to its high specific surface area, good microstructure, and excellent pore size distribution when compared with those of CuS/C-80 and CuS/C-160. Therefore, the experimental temperature was the key factor that affected the performance of the initial electrode material prior to the formation of the composite, implying that when combined with PANI, CuS/C-120@PANI is an extremely promising material that will hopefully be widely used in high-performance battery materials.

## Figures and Tables

**Table 1:** XRD analyses of the CuS/C-120 material (CuS-peaks). FWHM: full width at half maximum.

No.	2θ°	FWHM	Area (cts × 2θ°)	d-Spacing (Å)	Height (cts)	Rel. Int. (%)	HKL
1	27.738	2.473	75,853	3.2204	578	37.2	101
2	29.323	0.842	50,740	3.0524	1134	24.9	102
3	31.914	1.948	203,710	2.8050	1670	100	103
4	38.915	2.550	15,645	2.3132	15,645	7.7	105
5	48.016	0.774	113,625	1.8963	2049	55.8	110
6	52.777	2.352	34,255	1.7383	249	16.8	108
7	59.428	1.947	73,385	1.5635	564	36.0	116

**Table 2:** The value of IR drops at different current densities for CuS/C-120@PANI electrode. PANI: polyaniline.

**Current Density (Ag^−1^)**	0.5	1	2	3	4	5
**IR Drop (V)**	0.03	0.05	0.11	0.15	0.20	0.26
